# KIF20A Affects the Prognosis of Bladder Cancer by Promoting the Proliferation and Metastasis of Bladder Cancer Cells

**DOI:** 10.1155/2019/4863182

**Published:** 2019-04-09

**Authors:** Tianyu Shen, Long Yang, Zheng Zhang, Jianpeng Yu, Liang Dai, Minjun Gao, Zhiqun Shang, Yuanjie Niu

**Affiliations:** ^1^Tianjin Institute of Urology, The Second Hospital of Tianjin Medical University, Pingjiang Rd 23#, Hexi District, Tianjin 300211, China; ^2^Tianjin Medical University General Hospital, Tianjin, China; ^3^Department of Public Health, Saint Louis University, MO 63103, USA

## Abstract

**Objective:**

To investigate the expression of kinesin family member 20A (KIF20A) in bladder cancer, the effect of KIF20A on the proliferation and metastasis of bladder cancer cells, and the effect of KIF20A expression on the prognosis of bladder cancer patients.

**Methods:**

Bladder cancer tissue and its adjacent tissues were collected from tumour patients. The mRNA and protein expression levels of KIF20A in the tissue samples were detected by qRT-PCR and western blot. Immunohistochemical (IHC) staining was used to identify the expression and distribution of KIF20A proteins in the tissue samples. The relationship between the KIF20A expression and the clinical pathology of bladder cancer was analysed. The effect of the differential expression of KIF20A on the prognosis of patients with bladder cancer was analysed by the TCGA database. The plasmid was transfected into the bladder cell lines T24 and 5637 to construct two stable cell lines with knocked down KIF20A. The effect of KIF20A expression on the proliferation and invasion of T24 and 5637 bladder cells was explored in vitro using the abovementioned stable cell lines. The effect of the KIF20A expression on the proliferation of bladder cancer cells was evaluated by a mouse xenograft model.

**Results:**

The expression of KIF20A was significantly higher in the bladder cancer tissues than in the adjacent control tissues. The expression of KIF20A was significantly associated with the degree of pathological differentiation of bladder cancer. Patients with a higher expression of KIF20A had a higher tumour grade and a more advanced stage. The mean survival of patients with a high KIF20A expression was significantly lower than the mean survival of patients with a low KIF20A expression. The in vitro experiments demonstrated that the knockdown of KIF20A significantly inhibited T24 and 5637 cell proliferation and invasion. The in vivo experiments showed that the knockdown of KIF20A significantly inhibited the proliferation of the bladder tumours.

**Conclusion:**

KIF20A promotes the proliferation and metastasis of bladder cancer cells. Bladder cancer patients with a high KIF20A expression have a worse tumour differentiation and a poor prognosis. KIF20A may become an independent factor that affects the prognosis of bladder cancer patients and a therapeutic target for bladder cancer.

## 1. Introduction

Bladder cancer is one of the most common malignant tumours in the urinary system. According to cancer statistics, the estimated number of new bladder cancer cases increased from 79030 in 2017 to 81190 in 2018 in the United States. The number of deaths also increased from 16870 to 17240 [1, 2]. Treating bladder cancer is often difficult and expensive [3]. In recent years, bladder cancer morbidity and mortality have increased in the Chinese population. The time of diagnosis plays a crucial role in achieving a good prognosis [4]. Current chemotherapy methods and surgery can effectively prolong the survival of patients with bladder cancer, and patients need to bear the high expenses and pain caused by surgical treatment. Therefore, finding molecular markers that are potential therapeutic targets and prognostic indicators of bladder cancer is critical for a clinically accurate diagnosis and treatment.

The KIF family of molecules possesses a highly conserved kinetic domain, and many of its family members have ATP activity and are able to move towards the positive pole of the microtubule [5–9]. These molecules are involved in various physiological functions, such as intracellular spindle formation, chromosome partitioning, and substance transport [5, 8, 9]. Kinesin family member 20A (KIF20A), also known as MKLP2 and RAB6KIFL, is located on chromosome 5q31.2 [5]. The encoded protein contains 890 amino acids and has a molecular weight of approximately 100 kDa [10]. KIF20A mainly accumulates in the central region of the mitotic cell spindle and participates in the process of cell mitosis [11]. Studies have found that KIF20A is highly expressed in many types of tumours, such as lung cancer [12, 13], breast cancer [14], gastric cancer [15], liver cancer [16], bladder cancer [17], and pancreatic cancer [18–20]. Taniuchi et al. found that the KIF20A levels are elevated in pancreatic cancer [21]. If the expression of KIF20A is downregulated, there is a significant reduction in the proliferation of pancreatic cancer cells. In recent years, some scholars have pointed out that the downregulation of KIF20A can induce gastric cancer cell mitosis (G2/M phase) arrest and enhance chemotherapy drug sensitivity [15]. At present, research on the effect of KIF20A on the proliferation, invasion, and migration of bladder cancer cells is still in the preliminary stage, and the specific regulation and mechanisms of KIF20A have yet to be studied.

In this study, we examined the expression of KIF20A in clinical specimens of bladder cancer and found that the expression of KIF20A in the bladder cancer tissues is higher than that in the adjacent tissues. We further analysed the relationship between the KIF20A expression and the clinical pathology of bladder cancer. Statistical results showed that patients with a higher expression of KIF20A had a higher tumour grade and a more advanced stage. The effect of the differential expression of KIF20A on the prognosis of patients with bladder cancer was analysed by the TCGA database. The effects of KIF20A on the proliferation and invasion of bladder cancer cells were detected in vitro and in vivo.

## 2. Materials and Methods

### 2.1. Antibodies

Antibodies for the following proteins were used in this study for western blot and immunohistochemistry: KIF20A (Abcam, ab104118, 1 : 1000 dilution for western blot and 1 : 200 dilution for IHC-P), PCNA (Abcam, ab92552, 1 : 1000 dilution for western blot), Ki67 (Abcam, ab16667, 1 : 1000 dilution for western blot), Bcl-2 (Abcam, ab32124, 1 : 1000 dilution for western blot), caspase-3 (Abcam, ab13847, 1 : 500 dilution for western blot), MMP-2 (Abcam, ab37150, 1 : 500 dilution for western blot), and GAPDH (Sungene Biotech, KM9002, 1 : 5000 dilution for western blot).

### 2.2. Cell Culture and Cell Lines

The cell lines involved in this experiment, including T24, 5637, EJ, BIU87, and SV-HUC-1, were purchased from ATCC. These cells were cultured in RPMI 1640 medium (Gibco, Waltham, MA, USA) containing 10% foetal bovine serum (Gibco, Waltham, MA, USA) with culture conditions of 37.0°C with 5% CO_2_.

### 2.3. MTT Assay

The MTT powder was formulated into a solution at a concentration of 5 g/mL. The cells were seeded in a 96-well plate and incubated for 3-6 days at 37°C with 5% CO_2_ in a cell culture incubator. Then, 50 *μ*L of MTT solution was added to each well and incubated at 37°C for 4 hours. The supernatant was aspirated, and 150 *μ*L of DMSO was added to each well. A microplate reader measured the optical density (OD) value of each well at a wavelength of 490 nm.

### 2.4. Western Blot

Total cellular protein was extracted. The prepared protein samples were added to the corresponding gel lane of the separation gel, and electrophoresis was carried out using a constant voltage. Protein transfer was performed using a PVDF membrane. After the completion of the electroporation, the PVDF membrane was sealed with skim milk for 60 minutes. After washing the gel with TBST, the primary antibody was added and the membrane was incubated overnight at 4°C. The primary antibody was washed away by TBST; the secondary antibody solution corresponding to the primary antibody was added, and the membrane was incubated at room temperature for 1 hour. After washing away the secondary antibody with TBST, the membrane was prepared for exposure. The exposure reagents A and B were mixed in equal proportion; the mixed solution was applied to the PVDF membrane, and the membrane was exposed.

### 2.5. Immunohistochemistry

The paraffinized tissue sections were dewaxed in water and subjected to antigen retrieval. Briefly, 3% H_2_O_2_ was added to the sections, and the sections were incubated for 15 minutes at room temperature. After washing with PBS, the primary antibody was added dropwise and the sections were incubated at 4°C for 18 hours. After washing with PBS, the secondary antibody was applied to the specimens and the specimens were incubated at 37°C for 1 hour. The sections were stained with DAB solution. After washing with tap water, haematoxylin was added to the specimen to counterstain the cell nuclei. After washing with tap water, the sections were dehydrated, a transparent coverslip was mounted, and the slides were sealed. The results were observed under a microscope, and the positive staining rate was counted.

### 2.6. Colony Formation Assay

The cells were seeded in a culture dish and cultured in the abovementioned manner. The culture was terminated when macroscopic colonies appeared in the culture dish. The supernatant was discarded, and the cells were washed 3 times with PBS. The cells were fixed for 15 minutes, and an appropriate amount of Giemsa staining solution was added for 10 to 30 minutes. The number of colonies was then counted.

### 2.7. Transwell Invasion Assay

Matrigel was stored at 4°C overnight. Matrigel was diluted with prechilled RPMI 1640 medium, and 60 *μ*L of the diluted gel was added to each Transwell chamber of a 24-well plate for 2 hours. Cells were plated in each Transwell chamber. The serum-containing medium was added to the lower Transwell chamber surface. The cells were cultured for 24 hours with the abovementioned cell culture method. The Transwell chambers were removed, and cell fixation, staining, and counting were performed.

### 2.8. In Vitro Transfection

The following shRNA plasmids were used in this study for in vitro transfection: KIF20A human shRNA plasmid (CAT#: TG311916, OriGene) and HuSH shRNA RFP cloning vector (CAT#: TR30014, OriGene). The cells were transfected with liposomes. The plasmid was mixed with the transfection reagent at a ratio of 1 : 1-1 : 4, and the mixture was added to Opti-MEM for 30 minutes. The above mixture was then added to the medium with the cells. The medium was replaced with new medium after 24-48 hours. The transfected cells were screened using G418. A stably transfected cell line was finally obtained.

### 2.9. RNA Isolation and Quantitative RT-PCR Analysis

Total RNA was extracted using the TRIzol reagent (Invitrogen) according to the manufacturer's protocol. The RNA was reverse transcribed using a reverse transcription kit to obtain cDNA. The mRNA reverse transcription-PCR (RT-PCR) primers for KIF20A and GAPDH were purchased from Applied Biosystems. The primers were designed as follows: for KIF20A, forward primer, 5′-TGCTGTCCGATGACGATGTC-3′, reverse primer, 5′-AGGTTCTTGCGTACCACAGAC-3′; and for GAPDH, forward primer, 5′-AGGTTCTTGCGTACCACAGAC-3′, reverse primer, 5′-GCCATCACGCCACAGTTTC-3′. The expression of the mRNAs was determined in quantitative RT-PCR with an Applied Biosystems 7900 Real-Time PCR System (Thermo Scientific, Waltham, MA, USA). Small nucleolar RNA U6 was used as an internal reference for normalization.

### 2.10. Statistical Analysis

Statistical processes were performed with SPSS 20.0. Multiple groups were compared using one-way analysis of variance. The LSD test was used for comparisons between groups. Comparisons between the different treatment groups and control groups were performed using paired *t*-tests. Data analysis was performed with GraphPad Prism 5. *P* < 0.05 indicated a statistically significant difference in the results. *P* < 0.05 was marked as ∗, *P* < 0.01 was marked as ∗∗, *P* < 0.01 was marked as ∗∗∗, and no significant difference was expressed by “n.s.”

## 3. Results

### 3.1. KIF20A Expression Is Upregulated in Bladder Cancer

**Figure 1:**
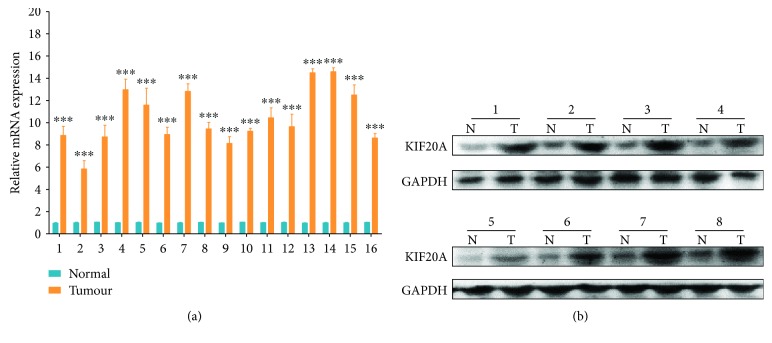
KIF20A is upregulated in bladder cancer tissues. (a) qRT-PCR was used to detect the expression level of KIF20A mRNA in surgical specimens of bladder cancer and its adjacent tissues from tumour patients. (b) Western blot was used to detect the protein expression level of KIF20A in the abovementioned specimens.

To study the expression of KIF20A in gastric cancer, the research team collected 16 surgical specimens of bladder cancer and their adjacent tissues from tumour patients. We first used qRT-PCR to detect the mRNA expression level of KIF20A in the above samples (Figure 1(a)) and found that the mRNA expression level of KIF20A was higher in the bladder cancer tissues than in the adjacent tissues of 16 sample pairs. Subsequently, we randomly selected 8 pairs of the 16 pairs of samples to detect the protein expression level of KIF20A. Western blot results showed that the expression level of the KIF20A protein was higher in the tumour tissue than in the adjacent tissues (Figure 1(b)). The preliminary results indicated that both the transcriptional and translational KIF20A expression levels were increased in bladder cancer.

### 3.2. High Expression of KIF20A Suggests a High Degree of Malignancy and a Poor Prognosis in Bladder Cancer

**Figure 2:**
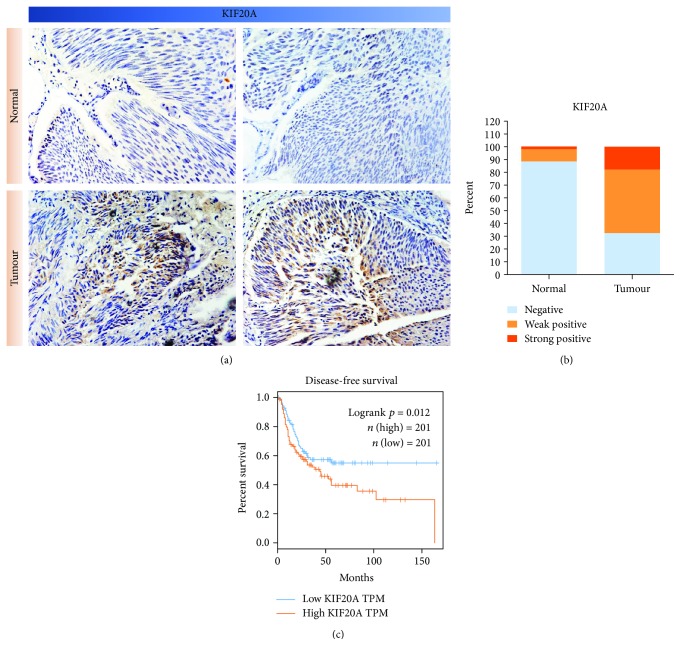
High KIF20A expression suggests a poor prognosis in bladder cancer. (a) Representative IHC staining images of KIF20A in bladder cancer and its adjacent tissues. (b) Chart of positive immunohistochemical rates and the associated statistics. (c) Survival curves for patients with bladder cancer (*n* = 402, *P* = 0.012) that were mapped with the website http://gepia.cancer-pku.cn/.

To further explore the relationship between the expression of KIF20A and the malignancy of bladder cancer, the research team performed immunohistochemical staining on 108 pairs of paraffinized bladder cancer and adjacent tissue sections (Figure 2(a)). The results showed that KIF20A was mainly expressed in the cytoplasm and membranes. According to the results of the immunohistochemical scoring, the positive rate of the KIF20A expression in bladder cancer tissues was 67.6% (17.9% strong positive, 49.7% weak positive) (Figure 2(b)). The positive rate in the adjacent tissues was 11.7% (2.2% strong positive, 9.5% weak positive) (Figure 2(b)). These results confirmed that the expression of KIF20A in the bladder cancer tissues was significantly higher than that in the adjacent tissues and the difference was statistically significant (*P* < 0.05). This result again confirms the conclusion from Figure 1. We collected essential and tumour status information from 108 patients with bladder cancer.  Table 1 showed that patients with a higher expression of KIF20A had a higher tumour grade and a more advanced stage. Additionally, lymph node metastasis and vascular invasion of the tumours were also associated with a high KIF20A expression.

We used the GEPIA website (http://gepia.cancer-pku.cn/) [22] to examine the effect of the KIF20A expression on patient survival. These data are from The Cancer Genome Atlas (TCGA) database. The survival rate of patients with bladder cancer with a high KIF20A expression was significantly lower than that of patients with bladder cancer with a low KIF20A expression (*P* = 0.012) (Figure 2(c)). This suggests that the high KIF20A expression indicates a poor prognosis in patients with bladder cancer. KIF20A can be an independent factor that affects the prognosis of patients with bladder cancer.

### 3.3. Knockdown of KIF20A Inhibits the Proliferation and Invasion of Bladder Cancer Cells

**Figure 3:**
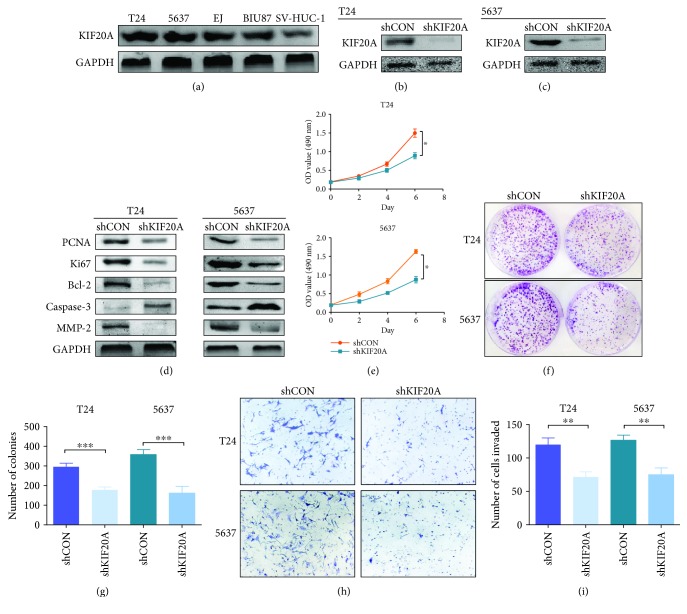
Knockdown of KIF20A inhibits the proliferation and invasion of bladder cancer cells. (a) Western blot was used to detect the protein expression levels of KIF20A in T24, BIU87, EJ, 5637, and SV-HUC-1 cells. (b, c) The shKIF20A plasmid was transfected into T24 and 5637 cells. The protein expression levels of KIF20A after transfection were detected by western blot. (d) Western blot was used to detect the expression levels of PCNA, Ki67, Bcl-2, caspase-3, and MMP-2 after the knockdown of KIF20A in T24 cells. (e) The MTT assay detected the growth of bladder cancer cells after the knockdown of KIF20A. The absorbance value was detected at a wavelength of 490 nm (^∗^*P* < 0.05). (f) A cloning formation assay detected the growth of bladder cancer cells after the knockdown of KIF20A. (g) The number of colonies in (g) was counted and plotted on a graph (^∗∗∗^*P* < 0.001). (h) Transwell invasion assays detected the invasiveness of the bladder cancer cells after the knockdown of KIF20A. (i) The number of invaded cells in (h) was counted and plotted on a graph (^∗∗^*P* < 0.01).

We further explored the effect of KIF20A on the biological function of bladder cancer cells in vitro. First, we used western blot to detect the protein expression levels of KIF20A in the bladder cancer cell lines T24, BIU87, EJ, and 5637 and in the normal bladder cell line SV-HUC-1. The results showed that the protein levels of KIF20A in the bladder cancer cell lines were higher than those in the normal bladder cell line (Figure 3(a)). This is consistent with the results shown in Figure 1. Since the protein expression level of KIF20A was higher in T24 and 5637 cell lines than in the other bladder cancer cell lines, we chose these two cell lines for experimental studies. We transfected T24/5637 cells with the shKIF20A plasmid and screened them to obtain the stable T24/5637 knockdown cell lines T24shKIF20A/5637shKIF20A. The knockdown efficiency was again detected by western blot (Figures 3(b) and 3(c)). It has been reported that KIF20A is involved in cell proliferation, apoptosis, and even metastasis [13, 23, 24], so we tested the corresponding indicators in the abovementioned stable cell lines. We found that the expression levels of PCNA and Ki67 in T24shKIF20A/5637shKIF20A were lower than those in the control group (Figure 3(d)). That is, the proliferation of bladder cancer cells is inhibited after knocking down KIF20A. At the same time, the expression level of Bcl-2 in T24shKIF20A/5637shKIF20A was lower than that in the control group (Figure 3(d)), and the expression level of caspase-3 was higher in T24shKIF20A/5637shKIF20A than in the control group (Figure 3(d)). These data indicate that the knockdown of KIF20A effectively promoted apoptosis in the bladder cancer cell lines T24/5637. Moreover, the expression of MMP-2 in T24shKIF20A/5637shKIF20A was also lower than that in the control group (Figure 3(d)), indicating that the invasive ability of the cells was also inhibited compared with that of the control group. The MTT assay further tested the effect of knocking down KIF20A on the proliferation of the bladder cancer cell lines T24/5637. The results showed that the proliferation ability of T24shKIF20A/5637shKIF20A was weaker than that of the control group (Figure 3(e)). The results of the colony formation assay showed that the number of cell colonies in the T24shKIF20A/5637shKIF20A group was significantly lower than that in the control group (Figures 3(f) and 3(g)), which confirmed that the knockdown of KIF20A could effectively inhibit the proliferation of bladder cancer cells. Transwell invasion assays were performed to detect the effect of KIF20A on the invasion of bladder cancer cells, and the results showed that the number of invading cancer cells in the T24shKIF20A/5637shKIF20A group was significantly lower than that in the control group (Figures 3(h) and 3(i)). We believe that knocking down KIF20A can effectively reduce the invasion ability of bladder cancer cells.

### 3.4. KIF20A Promotes the Growth of Bladder Tumours In Vivo

**Figure 4:**
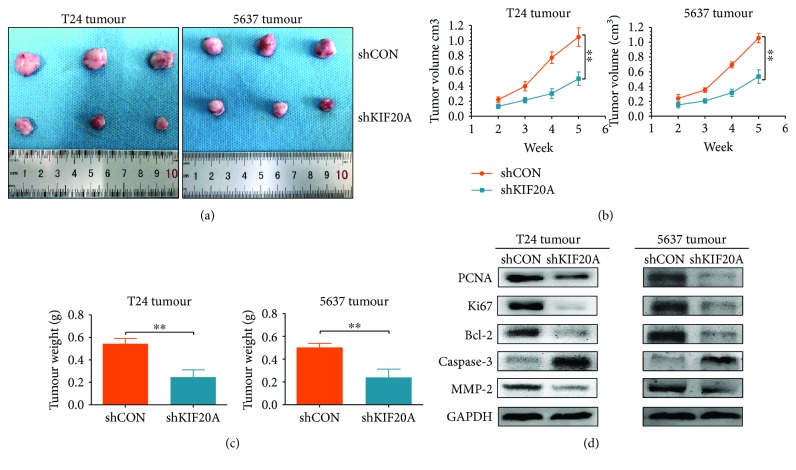
KIF20A promotes the growth of the bladder tumours in vivo. (a) The tumours were compared between the shKIF20A and shCON groups. (b) Subcutaneous T24 xenograft tumour volumes were compared between the shKIF20A and shCON groups (^∗∗^*P* < 0.01). (c) The tumour weights were compared between the shKIF20A and shCON groups (^∗∗^*P* < 0.01). (d) Western blot analysis of PCNA, Ki67, Bcl-2, caspase-3, and MMP-2 expression levels in the tumours of the shKIF20A and shCON groups.

The abovementioned two cell lines were used to establish xenograft tumour models. We divided 12 nude BALB/C mice into four groups on average, and each group was implanted with the following cell lines: T24, T24shKIF20A, 5637, and 5637shKIF20A. The size of the tumour was measured with the Vernier calipers two weeks after the inoculation and then measured once a week. The tumours were removed at week 5, and each tumour was weighed. Based on the tumour photograph (Figure 4(a)) and tumour growth curve (Figure 4(b)), the tumour volume of the shKIF20A group was significantly smaller than that of the shCON group. The tumour weight of the shKIF20A group was also lower than that of the shCON group (Figure 4(c)). We extracted total protein from the tumour tissues and examined the indicators for proliferation, apoptosis, and metastasis by western blot (Figure 4(d)). The results showed that the proliferation and metastatic ability of the tumours in the shKIF20A group were weaker than those of the tumours in the control group. The in vivo experiments confirmed that tumour growth was significantly inhibited after knocking down KIF20A.

## 4. Discussion

Because the diagnosis and treatment of bladder cancer is difficult, it is especially important to find a biomarker for the early diagnosis of bladder cancer and as a target for treatment. Based on our knowledge of the current scientific research, this is the first report on KIF20A in bladder cancer. We confirmed that KIF20A promotes the proliferation and metastasis of bladder cancer cells. Bladder cancer patients with a high KIF20A expression have a worse tumour differentiation and a poor prognosis. More importantly, KIF20A may become an independent factor that affects the prognosis of bladder cancer patients and a therapeutic target for bladder cancer.

Kinesin family member 20A (KIF20A) is also known as mitotic kinesin-like protein 2 (MKLP2) [5]. As a member of the kinesin-6 subfamily, KIF20A is a microtubule-associated motor protein. KIF20A is involved in the transport of organelles or cell membranes, as well as in activities such as cell division [24–26]. KIF20A is also involved in the formation of the spindle [8, 13]. It has been reported that the mitosis of cells could be regulated by KIF20A [27, 28]. The abnormal expression of KIF20A may lead to abnormal cell division, which can then lead to chromosomal aneuploidy and genomic instability in cancer [29–31]. In 2005, scientists first discovered that KIF20A is overexpressed in pancreatic cancer and silenced KIF20A with siRNA to inhibit the growth of pancreatic cancer cells [21]. In a related study of liver cancer, Lu et al. reported that KIF20A might be an independent factor that predicts overall survival and recurrence-free survival in patients with hepatocellular carcinoma [32]. These conclusions are consistent with our experimental results. In addition, KIF20A is highly expressed in glioma cell lines and glioma tissues. Patients with gliomas and a high KIF20A expression have a poor prognosis [33]. Studies have even shown that KIF20A also plays an essential role in the resistance to traditional chemotherapy drugs, such as paclitaxel. The high expression of KIF20A leads to paclitaxel resistance in breast cancer cell lines [34]. The molecular mechanism of KIF20A in cancer is still unclear. It has been reported that FOXM1 can enhance the radioresistance of lung cancer by inducing the expression of KIF20A [35].

Many studies have found that the upregulation of KIF20A is associated with cancer, but the development and potential molecular mechanisms of KIF20A in bladder cancer are not well understood. We studied the relationship between the expression of KIF20A and the clinicopathological features and prognosis of bladder cancer. The final results showed that the tumour differentiation of patients with a high KIF20A expression was worse than that of patients with a low KIF20A expression. According to the analysis of TCGA data, the high KIF20A expression in bladder cancer patients leads to a decrease in disease-free survival. Because KIF20A is closely related to cell division, a significant feature of malignant tumours is uncontrolled cell growth. Based on the above information, we hypothesized that KIF20A could affect the proliferation of bladder cancer cells. To demonstrate this hypothesis, we confirmed in vitro and in vivo that the high expression levels of KIF20A indeed promote the proliferation of bladder cancer cells.

In summary, KIF20A is likely to be a potential target for cancer therapy in bladder cancer. This finding will help in the development of antibladder cancer drugs. Our research had certain limitations. Due to the lack of follow-up of clinical patients in our hospital, we were unable to perform survival analyses. In subsequent work, we will expand the sample size. The mechanism by which KIF20A promotes the proliferation and metastasis of bladder cancer cells has not been studied in depth. Our group will continue to explore the molecular mechanism of KIF20A in the development of bladder cancer.

## Figures and Tables

**Table 1:** Relationships of KIF20A and the clinicopathological characteristics of 108 patients with bladder cancer.

Variables	All *n* = 108	KIF20A		*P* value^#^
		Low *n* = 35	High *n* = 73	
Age				
<65	56	17	39	0.64
≥65	52	18	34	
Sex				
Male	65	20	45	0.65
Female	43	15	28	
Tumour stage				
T2	54	23	31	0.02^∗^
T3/T4	54	12	42	
Tumour grade				
Low	64	34	30	0.01^∗^
High	54	11	43	
Lymph node metastasis				
No	45	20	25	0.02^∗^
Yes	63	15	48	
Distant metastasis				
No	60	20	40	0.82
Yes	48	15	33	
Vascular invasion				
No	54	23	31	0.02^∗^
Yes	54	12	42	

^#^ *P* value was analysed by a chi-square test; ∗ indicates *P* < 0.05 with statistical significance.

## Data Availability

The data used to support the findings of this study are available from the corresponding authors upon request.
