# Correction to “KIF20A Affects the Prognosis of Bladder Cancer by Promoting the Proliferation and Metastasis of Bladder Cancer Cells”

**DOI:** 10.1155/dim/9804137

**Published:** 2026-03-02

T. Shen, L. Yang, Z. Zhang, J. Yu, L. Dai, M.Gao, Z. Shang, and Y. Niu. “KIF20A Affects the Prognosis of Bladder Cancer by Promoting the Proliferation and Metastasis of Bladder Cancer Cells," *Disease Markers*, no. 2019 (2019), https://doi.org/10.1155/2019/4863182.

In the article titled “KIF20A Affects the Prognosis of Bladder Cancer by Promoting the Proliferation and Metastasis of Bladder Cancer Cells,” there was an error in Table 1 related to the incorrect presentation of patients with “low” tumor grading. The error was introduced by the authors during the preparation of the manuscript and should be corrected as follows:

**Table 1:** Relationships of KIF20A and the clinicopathological characteristics of 108 patients with bladder cancer.

Variables	All *n* = 108	KIF20A		*p* value^#^
		Low	High	
		*n* = 35	*n* = 73	
Age				
<65	56	17	39	0.64
≥65	52	18	34	—
Sex				
Male	65	20	45	0.65
Female	43	15	28	—
Tumor stage				
T2	54	23	31	0.02 ^∗^
T3/T4	54	12	42	—
Tumor grade				
Low	54	24	30	0.01 ^∗^
High	54	11	43	—
Lymph node metastasis				
No	45	20	25	0.02 ^∗^
Yes	63	15	48	—
Distant metastasis				
No	60	20	40	0.82
Yes	48	15	33	—
Vascular invasion				
No	54	23	31	0.02 ^∗^
Yes	54	12	42	—

^#^ *p* value was analyzed by chi‐square test;  ^∗^ indicates *p* < 0.05 with statistical significance.

We apologize for this error.

